# Fenton chemistry and oxidative stress mediate the toxicity of the β-amyloid peptide in a *Drosophila* model of Alzheimer’s disease

**DOI:** 10.1111/j.1460-9568.2009.06701.x

**Published:** 2009-04

**Authors:** Thomas Rival, Richard M Page, Dhianjali S Chandraratna, Timothy J Sendall, Edward Ryder, Beinan Liu, Huw Lewis, Thomas Rosahl, Robert Hider, L M Camargo, Mark S Shearman, Damian C Crowther, David A Lomas

**Affiliations:** 1 Department of Medicine, University of Cambridge, Cambridge Institute for Medical Research, Wellcome Trust/MRC BuildingHills Road, Cambridge CB2 0XY, UK; 2 Department of Genetics, University of CambridgeDowning Street, Cambridge CB2 3EH, UK; 3 The Neuroscience Research Centre, Merck Sharp & DohmeHarlow, Essex, UK; 4 Merck Research Laboratories, Merck & Co.Rahway, NJ, USA; 5 Department of Chemical Biology, Pharmaceutical Science Research Division, King’s College LondonLondon, UK; 6 Merck Research Laboratories, Merck & Co.Boston, MA, USA

**Keywords:** aggregation, chelation therapy, hydroxy radical, iron

## Abstract

The mechanism by which aggregates of the β-amyloid peptide (Aβ) mediate their toxicity is uncertain. We show here that the expression of the 42-amino-acid isoform of Aβ (Aβ_1–42_) changes the expression of genes involved in oxidative stress in a *Drosophila* model of Alzheimer’s disease. A subsequent genetic screen confirmed the importance of oxidative stress and a molecular dissection of the steps in the cellular metabolism of reactive oxygen species revealed that the iron-binding protein ferritin and the H_2_O_2_ scavenger catalase are the most potent suppressors of the toxicity of wild-type and Arctic (E22G) Aβ_1–42_. Likewise, treatment with the iron-binding compound clioquinol increased the lifespan of flies expressing Arctic Aβ_1–42_. The effect of iron appears to be mediated by oxidative stress as ferritin heavy chain co-expression reduced carbonyl levels in Aβ_1–42_ flies by 65% and restored the survival and locomotion function to normal. This was achieved despite the presence of elevated levels of the Aβ_1–42_. Taken together, our data show that oxidative stress, probably mediated by the hydroxyl radical and generated by the Fenton reaction, is essential for Aβ_1–42_ toxicity *in vivo* and provide strong support for Alzheimer’s disease therapies based on metal chelation.

## Introduction

There is a growing consensus that smaller, soluble aggregates of the β-amyloid peptide (Aβ), rather than mature amyloid plaques, are the pathogenic species in Alzheimer’s disease (AD) (Lambert *et al.*, 1998; Walsh *et al.*, 2002). However, the mechanism by which these aggregates mediate their toxicity remains unclear. As Aβ is generated in the extracellular space, or more likely within the lumen of endocytic vesicles (Koo & Squazzo, 1994; Refolo *et al.*, 1995), its toxic effects may be mediated by membrane damage or by interactions with membrane-bound proteins. There is evidence that Aβ aggregates can degrade the electrical resistance of membranes (Kayed *et al.*, 2004; Demuro *et al.*, 2005), possibly by forming pores (Lashuel *et al.*, 2002), or alternatively they may interact with membrane receptors or even gain access to the cytoplasm. Membranes can also be damaged by the reactive oxygen species that are generated by Aβ aggregates in the presence of metals such as copper, zinc or iron (Bush, 2003). Subsequent pathological processes include mitochondrial damage (Abramov *et al.*, 2004), tau phosphorylation with consequent axonal transport dysfunction and the initiation of cell death (Kienlen-Campard *et al.*, 2002; Wei *et al.*, 2002; Jo *et al.*, 2004). However, until recently (Cao *et al.*, 2008) it has been impossible to take a global view to ask which biological processes are essential for the development of the disease and which are downstream consequences of neurotoxicity. Knowing which biological processes are directly involved in initiating AD will allow us to focus on those upstream targets that have the greatest therapeutic potential.

We have developed a model of AD that is based on the expression of the human Aβ in fly neurons by coupling it to an N-terminal secretion signal peptide (Crowther *et al.*, 2005). The Aβ_1–42_ but not the Aβ_1–40_ control accumulates in the brain and results in decreased lifespan and impaired locomotor performance. These phenotypes are more marked in flies expressing the E22G (Arctic) mutant of the Aβ_1–42_, which causes increased aggregation of Aβ and is responsible for early onset familial AD (Nilsberth *et al.*, 2001). Here, we use our *Drosophila* model of AD to identify the pathways and intermediates that are critical for Aβ-mediated toxicity *in vivo*.

## Materials and methods

### Drosophila stocks

The following stocks were generous gifts: *UAS-CAT* [catalase (CAT) upstream activating sequence (UAS) inducible transgene] (Anderson *et al.*, 2005), *UAS-SOD1* [CuZn-superoxide dismutase (SOD)1] (Anderson *et al.*, 2005), *UAS-SOD1-IR* (RNAi line for SOD1) (Missirlis *et al.*, 2003) and *UAS-mitSOD2* (Mn-SOD2) (Anderson *et al.*, 2005) from Professor John Phillips (Guelph, Canada), *UAS-Sniffer* (carbonyl reductase) (Botella *et al.*, 2004) from Professor Stephan Schneuwly (Regensburg, Germany) and *UAS-GST* [glutathione-S-transferase (GST) S1] (Whitworth *et al.*, 2005) from Dr Alex Whitworth (Sheffield, UK). We used previously characterized *UAS-CAT*, *UAS-SOD1* and *UAS-mitSOD2* stocks (Anderson *et al.*, 2005) as they increase enzymatic activity by up to 200%. Stocks of mutant *SOD1*^*n108*^ (Phillips *et al.*, 1995) and *elav*^*c155*^*-GAL4* were from the Bloomington stock centre (Indiana, USA). Flies carrying Aβ transgenes [Aβ_1–40_ (*Alz40.1*), Aβ_1–42_ (*Alz42.2* and *Alz42.3*) or *Arctic* Aβ_1–42_ (*AlzArc1*)] have been described previously (Crowther *et al.*, 2005; Luheshi *et al.*, 2007). The transgenes are each representative of six independent transgenic lines and each transgene drives similar levels of mRNA whether alone or in combination (such as *Alz42.2* + *Alz42.3*).

### DNA constructs

The cDNA for *Drosophila* ferritin 1 heavy chain (Fer1HC) was isolated from EST clone GH24060 (Berkley *Drosophila* Genome Project). This cDNA lacked the iron response element resulting in the expression of a constitutively active form of Fer1HC. The cDNA for *Drosophila* ferritin 2 light chain (Fer2LC) naturally lacks an iron response element and was isolated from the EST clone AT16780 (Berkley *Drosophila* Genome Project). Both cDNAs were inserted downstream of GAL4 UASs (*UAS-Fer1HC* and *UAS-Fer2LC*) in the pUAST plasmid by directional cloning following *EcoR*I and *Xho*I digestion.

### Affymetrix cDNA microarray

Individual samples were each hybridized to a *Drosophila* Genome GeneChip® Array following standard Affymetrix protocols. Affymetrix Microarray Suite 5 was used to generate signal values and detection calls. Probe level intensity data were adjusted for background, normalized and log transformed using the robust multichip average pre-processing method (Irizarry *et al.*, 2003) using Rosetta Resolver® 7.1.

Ratio data for each individual probe were created as follows. Aβ_1–40_ baseline samples were created for each time-point by pooling all replicates (see Supplementary material, Fig. S1a). The individual replicates for each test condition (Arctic Aβ_1–42_ and Aβ_1–42_) were then compared with the age-matched control Aβ_1–40_ pool. All statistical analyses were performed on log_10_ ratio data.

Gene expression differences were determined by one-way, error-weighted anova on ratio data by comparing Aβ_1–42_ and Arctic mutants with age-matched Aβ_1–40_ controls (factor = genotype, *n* = 4 per group). Genes were only considered in the analysis of over-represented biological themes if the differential expression was highly significant (*P*<0.01).

### Characterization of over-represented biological themes

Gene Ontology (Ashburner *et al.*, 2000) enrichment analysis was performed using the Gene Ontology tree machine (http://bioinfo.vanderbilt.edu/gotm/) (Zhang *et al.*, 2004). In summary, a hypergeometric test was performed to determine whether a particular biological process or molecular function was disproportionately represented in the set of genes that were differentially expressed as compared with all of the genes in the GeneChip® array. Where *n*= number of genes that are differentially expressed between two experimental conditions (e.g. between Arctic Aβ_1–42_ and Aβ_1–40_ on day 0), *N*= total number of genes on the array, *K*= number of genes on the array that belong to the category of interest and *k*= number of genes that belong to the category that are differentially expressed, the significance (*P*) of enrichment for a given category is determined by 
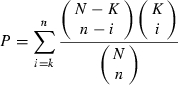
 A ratio of enrichment, *R*, is calculated as *R*= *k*/*k*_*e*_ where *k*_*e*_ = (*n*/*N*)*K* is the expected value for a given category if *n* = number of genes were a random sample of uniformly selected genes from the reference set of all genes on the chip.

### Gene Search element screen

A library of 3000 unique insertions of the Gene Search (GS) element was generated by mobilizing the GS element from the X chromosome (DGRC number 200079) to the autosomes by crossing with flies expressing the *Delta2-3* transposase. The first eclosed fly with a stably-jumped GS element from each mobilization cross was used to establish a GS line. Stocks were maintained by monitoring eye colour. The effect of the *Gal-4* activated GS element on the longevity of flies expressing the *Arctic* mutant of Aβ_1–42_ was determined by crossing male flies with *elav*^*c155*^*-Gal4* (*elav-Gal4*) on the X chromosome and the *Arctic* Aβ_1–42_ transgene on the second chromosome with virgin females with a floating GS element. All of the female offspring had both *elav-Gal4* and the *Arctic* Aβ_1–42_ transgene but only half had the GS element; the unmodified population (without the GS element) provided an internal control for each longevity assay. The null hypothesis was that the presence of the GS element made no difference to the longevity of the flies expressing the *Arctic* Aβ_1–42_ transgene.

Longevity assays for the primary screen were performed at 25°C, blind to the identity of the GS elements. Live flies were counted and the food changed on days 1, 3 and 5 of a 7 day cycle. A mean number of 17 flies was assessed per GS line (total number of flies assessed, 50 320; minimum number of flies assessed per GS line, 10). To detect suppression of the longevity phenotype we determined the time to 75% death, which is the median survival of the flies in which the GS element has prolonged their life. These survival times were normally distributed (*n* = 2893, mean 21.6 days, SD 3.8 days) and any lifespan that was more than two SDs greater than the mean was defined as significant.

In the secondary screen the chromosomal site of insertion was determined by classical genetics and homozygous GS flies were crossed so that all flies in the longevity assays expressed the activated GS element. This secondary screen used homozygous GS element stocks and assessed more than 30 flies, permitting robust comparison of the survival of GS-modified and unmodified *Arctic* Aβ_1–42_ flies. The site of the GS element insertion was identified in those lines with confirmed modifier activity by inverse polymerase chain reaction using the FlyChip facility (http://www.drosdel.org.uk/molecular_methods.php). The molecular function of genes with inserts within the coding sequence was determined from FlyBase. For inserts in non-coding DNA, the molecular function was determined for the genes on either side of the insert. The effect of the activated GS element on the survival of control flies and flies expressing the *AlzArc1* transgene was also determined. GS lines in which the modification of the Aβ longevity phenotype was not confirmed or in which the GS element had a marked non-specific effect on control flies were discarded.

### Longevity assays for the secondary screen and genetic modifier assessment

Longevity assays in the secondary screen and for each of the subsequent genetic modifiers followed at least 80 flies per genotype in groups of 10 flies per vial. Live flies were counted and their food changed on days 1, 3 and 5 of a 7 day cycle. For assessing the efficacy of metal chelation, clioquinol (Calbiochem) was dissolved in dimethylsulphoxide and the solution added to fly food to give a final concentration of 0.2% v/v. Survival curves were plotted using the Kaplan–Meier estimator. The statistical significance was calculated using the log rank test within the spss 11.0 statistical package. The null hypothesis in all of the longevity assays was that the presence of the GS element made no difference to the longevity of the flies expressing the *Arctic* Aβ_1–42_ transgene.

### Assay of sensorimotor performance

The sensorimotor performance of the flies was determined using a previously described negative geotaxis assay (Rival *et al.*, 2004). Fifteen flies were placed in a sterile plastic column (25 cm tall × 1.5 cm internal diameter) and tapped to the bottom. After 45 s the flies at the top of the column (*N*_top_) and the flies remaining at the bottom (*N*_bot_) were counted. Three trials were performed at 1 min intervals. The performance index was defined as (15 + *N*_top_–*N*_bot_)/30. Statistical analysis was performed using the two-tailed Student’s *t*-test.

### Quantitation of β-amyloid peptides

*Drosophila* were cultured at 25°C for 5 days after eclosion and then five heads were homogenized in 50 μL of 5 m guanidinium HCl, 5 mm EDTA and 50 mm hepes, pH 7.3. Following centrifugation for 5 min at 12 000 ***g***, 20 μL of the clear supernatant was removed and mixed with 180 μL of 25 mm HEPES, pH 7.3, 1 mm EDTA and 0.1% w/v bovine serum albumin with protease inhibitors (Complete™, Roche). Triplicate 25 μL aliquots were mixed with an equal volume of phosphate-buffered saline containing 2% w/v bovine serum albumin, 0.2% v/v Tween-20 and protease inhibitors (Complete™, Roche) in wells on a MESO microtitre plate (Standard bind, MA6000, no. P11AA-1; Meso Scale Discovery, MD, USA). The reaction was started by adding 25 μL of 4 μg/mL solutions of biotinylated 6E10 or 4G8 (Signet Laboratories, MA, USA) monoclonal antibodies. After mixing a further 25 μL aliquot of 1 μg/mL Ruthenium-labelled G2-10 or G2-11 (The Genetics Company, Switzerland), monoclonal antibody solution was added to each well. Following an overnight incubation at 25°C, the plates were washed twice with phosphate-buffered saline, 150 μL of S Read Buffer (R92SC-1, Meso Scale Discovery, MD, USA) was added and the measurement was taken in a Sector PR instrument (Meso Scale Discovery). Statistical analysis was performed using the two-tailed Student’s *t*-test.

### Hydrogen peroxide sensitivity assay

Two-day-old flies were cultured in a vial containing only filter paper soaked in an aqueous solution of 2% w/v sucrose and 10% v/v H_2_O_2_ or a control solution of 2% w/v sucrose alone. Ten vials of 15 flies were cultured for each genotype at 25°C with the soaked paper being replaced twice per day. Statistical analysis was performed using the two-tailed Student’s *t*-test.

### Carbonyl assay

Five adult flies (28 days old, grown at 25°C) were decapitated and the heads were immediately homogenized in water, sonicated and centrifuged at 12 000 ***g*** for 10 min and the clear supernatant assayed for protein content using the Bradford method. The protein concentration was adjusted to 5 μg/mL by the addition of phosphate-buffered saline. Protein carbonyl groups were assayed using an enzyme-linked immunosorbent assay-based protocol described by Alamdari *et al.* (2005). Protein carbonyl groups were reacted with dinitrophenol hydrazine (Sigma-Aldrich) and the resulting dinitrophenol adducts were detected using an anti-DNP rabbit polyclonal antibody (Sigma-Aldrich). Statistical analysis was performed using the two-tailed Student’s *t*-test.

### Iron and zinc determination in fly head extracts by mass spectrometry

Flies were cultured at 29°C and decapitated at 10 days old. Triplicates of 20 fly heads per condition were weighed by difference into a new 15 mL screw-top polypropylene centrifuge tube (part no. 2086-500, Elkay, UK). Nitric acid (500 μL) (‘Trace Select’, part no. 84385, Fluka, UK) was added and the tubes were sealed and incubated overnight at 65°C such that there was no visible solid matter remaining. The sample was prepared by resuspending the extracts in 5 mL of double-distilled water before analysis using an Elan 6100 DRC ICP/MS (Perkin-Elmer). The reaction cell was used for all measurements with the following operating conditions: 1.04 L/min nebulizer flow, 1100 W radio frequency power and a dynamic reaction cell gas flow of 0.7 mL/min of ammonia. Iron (Mr 55.9349), zinc (Mr 65.9260) and calcium (Mr 43.9555), but not copper (Mr 62.9298), were reliably detected. As calcium is not chelated by clioquinol it was used to control for the quantity of tissue in each sample.

## Results

### Microarray analysis of gene expression in flies expressing β-amyloid peptide supports the importance of oxidative stress in β-amyloid peptide toxicity

**Fig. 1:**
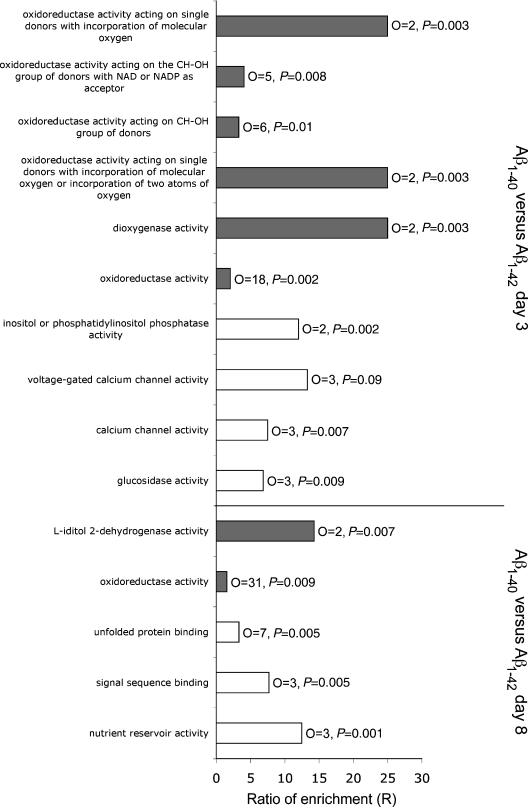
The expression signature of genes for flies expressing Aβ_1–42_ at day 3 (top part) and day 8 (bottom part) of adult life was determined and analysed for over-representation of genes belonging to particular molecular functions. Only functional groups that are significantly over-represented in the set of differentially regulated genes (*P* < 0.01) are depicted. The bars represent the ratio of enrichment (*R*), calculated as the ratio between the observed (*O*) number of genes belonging to a molecular function and the expected number of genes belonging to that function if selected at random. Molecular functions that are clearly redox-related are represented by shaded bars; other molecular functions are represented by open bars.

Affymetrix microarrays were used to identify gene expression signatures (one-way anova, *P* < 0.001) for flies expressing wild-type Aβ_1–42_ at day 0, 3 and 8 of adult life and *Arctic* Aβ_1–42_ at day 0 and 3 by comparison to age-matched Aβ_1–40_ controls. The derived gene expression signatures for flies expressing wild-type and *Arctic* Aβ_1–42_ were then used to determine the biological processes, molecular functions and cellular components that were over-represented in each set (in supplementary Fig. S1b). The role of redox genes appeared most apparent in the early stages of Aβ_1–42_ expression (Fig. 1); this is most clearly seen for flies expressing wild-type Aβ_1–42_ at day 3 (top part, shaded) where these genes comprised six of the 10 families of enriched genes. Genes that were classified as having ‘oxidoreductase activity’ were significantly enriched at both day 3 and 8 in flies expressing wild-type Aβ_1–42_ (*P* < 0.01). Similarly, carbonyl reductase (NADPH) activity was enriched in the day 3 signature of flies expressing *Arctic* Aβ_1–42_ (*P* < 0.01). Taken together, these observations provide strong support for the role of oxidative stress-related genes in the response of the fly brain to the expression of the Aβ.

### P-element screen for modifiers of β-amyloid peptide toxicity implicate redox regulation as an important target of modifier activity

**Fig. 2:**
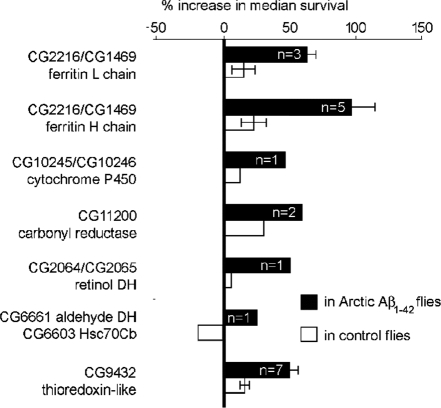
The percentage increase in median survival in flies expressing *Arctic* Aβ_1–42_ that was attributable to the GS element was calculated (filled bars). Control flies (*n* = 100) possessing *elav-Gal4*±the GS element but lacking the *Arctic* Aβ_1–42_ transgene (*n* = 100) were also assessed. The percentage increase in median survival in these control flies that was attributable to the GS element was determined (empty bars). The significance of the greater increase in median survival in *Arctic* Aβ_1–42_ vs. the effect on control flies was significant (*P* < 0.001) in all cases. The number of independent GS element inserts that were independently identified in the screen and analysed in this assay is indicated in white on the filled bars. Where three or more GS inserts were available in a particular class the error bars indicate the SD of the estimates. We assumed that the estimates varied normally and the significance was calculated by Student’s *t*-test [ferritin light (L) chain inserts, *P* < 0.05; ferritin heavy (H) chain inserts, *P* < 0.001; CG9432, *P* < 0.001]. DH, dehydrogenase.

A 3000-line library of unique GS (Toba *et al.*, 1999) element inserts was screened for lines that modified the longevity of *Drosophila* expressing Aβ_1–42_ in their central nervous system. The GS elements can either disrupt gene function when they insert within essential coding or non-coding DNA, or else they can bidirectionally upregulate neighbouring genes. In our screen 1.5% of the GS inserts resulted in an increase in median survival that was more than two SDs away from the mean, whereas 0.5% of the inserts significantly reduced survival. A secondary longevity assay was performed to confirm the initial findings but also to determine the effect of the GS elements on the survival of control flies that did not express Aβ. The insertions that specifically modified the longevity of Aβ-expressing flies, and not controls, were classified into 18 suppressor and three enhancer groups according to the identity of the neighbouring genes. Seven of these 21 classes were adjacent to genes with a predictable role in oxidative stress (Table 1 and Fig. 2). The selected suppressor GS elements increased median survival by 25–96% in flies expressing Aβ but had no, or little, effect in control flies (Fig. 2, filled and empty bars, respectively).

**Table 1:** GS elements inserted within or adjacent to genes involved in oxidative stress modify the toxicity of *Arctic* Aβ

GS elements	Gene name	Gene identifier	Molecular function	GS insertion sites (range of sequence locations)
148, 174, 274, 1360, 2225, 2823, 3063	Lethal (2) 01289	CG9432	Thioredoxin-like domain	2252526–2252617
2703, 2521, 3102	Fer1HC and Fer2LC	CG2216 and CG1469	Iron chelator and ferroxidase activity	26215006–26215015
532, 1287, 1472.2, 1652, 2484	Fer1HC and Fer2LC	CG2216 and CG1469	Iron chelator and ferroxidase activity	26212564–26212784
811	Cyp6a20 Cytochrome P450-6a9	CG10245 and CG10246	Oxidoreductase activity in steroid metabolism	10396945
1264, 2155	Carbonyl reductase	CG11200	Reduction of aldehydes	15823515–15823522
1357	Orthologues of human retinol dehydrogenase	CG2064 and CG2065	Short chain dehydrogenases	20961357
1774	Orthologue of human aldehyde dehydrogenase 4A1 and Hsc70Cb	CG6661 and CG6603	Oxidation of aldehydes and chaperone	14029850–14036659

Aβ, β-amyloid peptide; GS, Gene Search.

### Flies expressing β-amyloid peptide_1–42_ are more sensitive to oxidative stress and have higher levels of oxidative damage

**Fig. 3:**
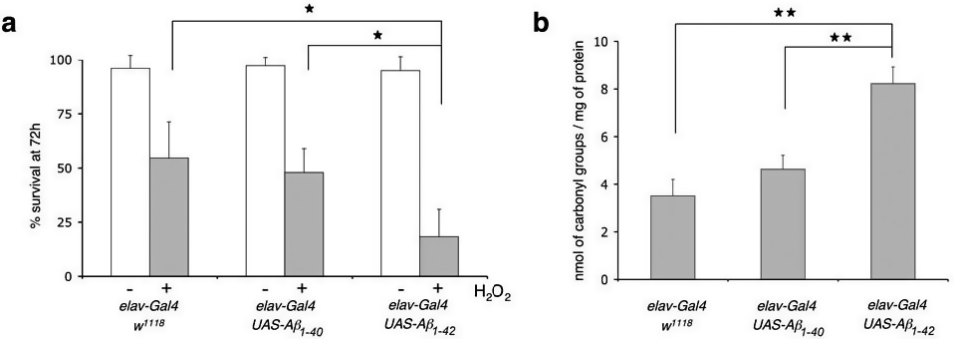
Flies expressing Aβ_1–42_ are more sensitive to oxidative stress and have higher levels of oxidative damage. Supplementing the fly food with 10% v/v H_2_O_2_ for 72:00 h (a, shaded bars) was specifically toxic to flies expressing Aβ_1–42_, significantly reducing their survival as compared with flies expressing Aβ_1–40_ (a, *elav-Gal4 UAS-Aβ*_*1–42*_ vs. *elav-Gal4 UAS-Aβ*_*1–40*_, *P* < 0.05). H_2_O_2_ treatment did not reduce the survival of Aβ_1–40_ as compared with control flies (a, *elav-Gal4 w*^*1118*^ vs. *elav-Gal4 UAS-Aβ*_*1–40*_). The differences in survival at 72:00 h were not due to toxicity of the Aβs because in the absence of H_2_O_2_ there was no difference in survival between any of the groups (open bars). Flies expressing Aβ_1–42_, but not flies expressing Aβ_1–40_ or control flies, exhibited markers of oxidative stress. Measurement of carbonyl groups (nm/mg head protein extract) demonstrated that flies expressing Aβ_1–42_ had a significantly higher carbonyl load than control flies (b, *elav-Gal4 w*^*1118*^ vs. *elav-Gal4 UAS-Aβ*_*1–42*_, *P* < 0.05). In flies expressing Aβ_1–40_ the carbonyl load was not significantly increased (b, *elav-Gal4 UAS-Aβ*_*1–40*_ vs. *elav-Gal4 w*^*1118*^) above control levels. The data are representative of three independent Aβ_42_ transgenic lines. Independent Student’s *t*-test result (*P < 0.05, **P < 0.01).

The genetic screen and microarray data implicated oxidative stress as playing a central role in the toxicity of the Aβ_1–42_. This was assessed by testing the response of flies to an oxidative insult in the form of H_2_O_2_ in their food. In these experiments we observed that flies expressing wild-type Aβ_1–42_ were more likely to die if their food was supplemented with 10% v/v H_2_O_2_ than either control flies (Fig. 3a, *elav-Gal4 w*^*1118*^ vs. *elav-Gal4 UAS-Aβ*_*1–42*_, *P* < 0.05) or flies expressing Aβ_1–40_ (Fig. 3a, *elav-Gal4 UAS-Aβ*_*1–40*_ vs. *elav-Gal4 UAS-Aβ*_*1–42*_, *P* < 0.05). When we quantified oxidative damage by measuring the levels of carbonyl groups in protein extracts of fly heads we found that flies expressing Aβ_1–40_ had carbonyl levels that were similar to those of control flies (Fig. 3b, *elav-Gal4 w*^*1118*^ vs. *elav-Gal4 UAS-Aβ*_*1–40*_), whereas flies expressing a single Aβ_1–42_ transgene had almost double the carbonyl levels of flies expressing Aβ_1–40_ (Fig. 3b, *elav-Gal4 UAS-Aβ*_*1–42*_ vs. *elav-Gal4 UAS-Aβ*_*1–40*_, *P* < 0.05).

### Transgenic over-expression of single antioxidative stress genes rescues the β-amyloid peptide-induced longevity phenotype

**Fig. 4:**
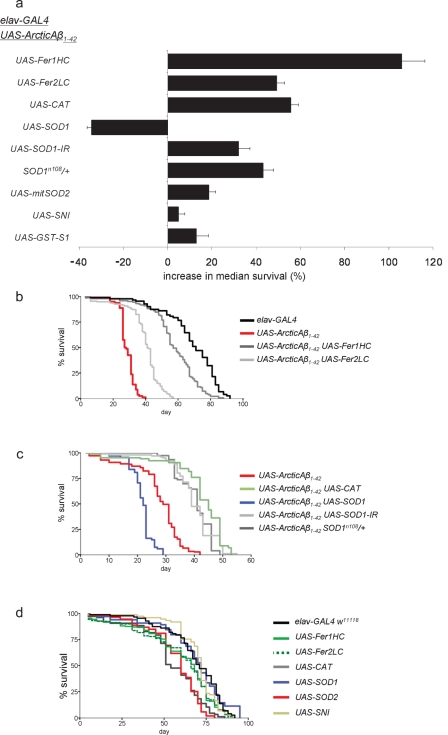
Co-expression of antioxidative stress genes with Aβ increased the median survival of flies. Co-expression of both ferritin heavy (*elav-Gal4 UAS-Fer1HC*) and light (*elav-Gal4 UAS-Fer2LC*) chains prolonged the lifespan of *Arctic* Aβ_1–42_ flies (a and b, *elav-Gal4 UAS-Arctic Aβ*_*42*_). Flies co-expressing *Arctic* Aβ_1–42_ and the heavy chain of ferritin exhibited survival that was similar to that of control flies (b, *elav-GAL4*). Similarly, co-expression of another gene from the GS screen, carbonyl reductase (a, *elav-Gal4 UAS-SNI*), yielded a small but significant prolongation of the lifespan of *Arctic* Aβ_1–42_ flies. The expression of other canonical antioxidative enzymes also had significant effects on longevity. Both mitSOD2 (a, *elav-Gal4 UAS-mitSOD2*) and CAT (a and c, *elav-Gal4 UAS-CAT*) prolonged the lifespan of the flies. Surprisingly, SOD1 (a and c, *elav-Gal4 UAS-SOD1*) enhanced the toxicity of *Arctic* Aβ_1–42_. In contrast, the knockdown of endogenous SOD1 protein by UAS-RNAi (a and c, *elav-Gal4 UAS-IR.SOD1*) protected the fly from Aβ toxicity. The enhancer effect of SOD1 appears to be mediated by its catalytic activity because a dominant negative mutant of SOD1 prolonged the lifespan of the flies expressing *Arctic* Aβ (a and c, *SOD1*^*n108*^). In control experiments there was no prolongation of lifespan when SOD1, mitSOD2, CAT, carbonyl reductase, ferritin heavy chain and ferritin light chain were expressed using *elav*^*c155*^*-Gal4* in flies that did not carry the *UAS-Arctic* Aβ_1–42_ transgene (d). Kaplan–Meier survival curves were plotted and statistical significance was assessed by the log rank test using the spss 11.0 statistical package. Differences shown were all statistically significant (*P* < 0.001). In control experiments *elav-Gal4* flies had the same lifespan as the background *w*^*1118*^ flies.

The ability of antioxidative stress genes identified in the screen, and genes from the canonical oxidative stress pathway, to modify the toxicity of Aβ was confirmed in flies by specifically over-expressing each transgene in combination with wild-type or *Arctic* Aβ_1–42_. In this way we assessed candidate genes from the GS element screen, i.e. the heavy and light chains of *Drosophila* ferritin (*Fer1HC* and *Fer2LC*) and carbonyl reductase by over-expression of the *Drosophila* enzyme *Sniffer* (SNI). We also tested candidate genes including cytoplasmic *CuZn-SOD1*, the mitochondrial *Mn-SOD2* (*mitSOD2*) and *CAT*, and *GST* (Fig. 4). This approach allowed comparison of the efficacy of the upstream enzymes that modulate the generation of free radicals (SOD1, mitSOD2, CAT, Fer1HC and Fer2LC) with the downstream enzymes that repair oxidative damage (GST and carbonyl reductase/SNI).

Over-expression of ferritin heavy chain resulted in a 105% increase in median survival of *Arctic* Aβ_1–42_ flies (*P* < 0.0001), whereas over-expression of ferritin light chain gave a 49% increase in median survival (*P* < 0.0001) (Fig. 4a and b). The most powerful canonical oxidative stress-related protein was CAT, which increased the median survival of *Arctic* Aβ_1–42_ flies by 56% (*P* < 0.0001, Fig. 4a and c). MitSOD2 prolonged median survival by 18% (*P* < 0.0001, Fig. 4a and d). In contrast, and contrary to our expectations, the over-expression of cytoplasmic SOD1 enhanced the toxicity of *Arctic* Aβ_1–42_. Flies expressing mutants of SOD1 that are dominant-negative for activity were then assessed to see if they had a similar modifying activity. Co-expression of both the SOD1^n108^ mutant and RNAi for SOD1 resulted in a 43% and 32% increase in median survival, respectively (Fig. 4a and c), indicating that it is the catalytic activity of SOD1 that potentiates the toxic effect of the Aβ. The protection afforded by CAT combined with the toxicity of SOD1 suggests that the uncompensated production of H_2_O_2_ is a vital step in the oxidative stress caused by the Aβ. An additional stress resulting from increased SOD1 activity may be the co-production of O_2_ by the dismutation of superoxide radicals. This oxygen may go on to generate further H_2_O_2_ by reacting with iron or copper ions that are complexed with Aβ (Huang *et al.*, 1999a,b;).

Much smaller protective activities were observed when enzymes, specifically *Drosophila* carbonyl reductase (SNI) and GST, that are involved in steps further downstream in the pathway were upregulated (Fig. 4a). Control experiments, in which the antioxidative stress genes were over-expressed in flies that were identical except that they lacked the Aβ transgene, did not reveal non-specific prolongation of life (Fig. 4d). In flies expressing wild-type Aβ_1–42_ we were able to confirm the prolongation of lifespan by the heavy and light chains of ferritin and the toxic effect of SOD1 (data not shown).

### Transgenic over-expression of single antioxidative stress genes rescues the β-amyloid peptide-induced locomotor phenotype

**Fig. 5:**
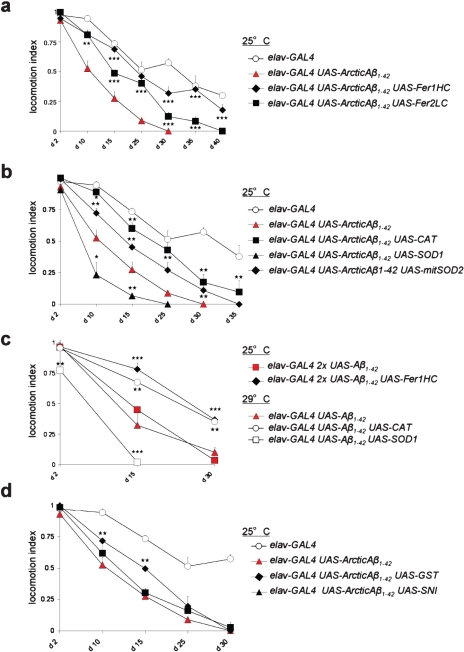
Over-expressing oxidative stress-related genes modified the Aβ-induced locomotor phenotype. Flies expressing *Arctic* Aβ_1–42_ (a, *UAS-Arctic Aβ*_*1–42*_) exhibited a progressive decline in locomotor function becoming immobile by day 30. Control flies (*elav-GAL4*) showed a markedly slower decline in locomotor function (a–d). Co-expression of ferritin heavy (a, *UAS-Arctic Aβ*_*1–42*_*UAS-Fer1HC*) and light (a, *UAS-Arctic Aβ*_*1–42*_*UAS-Fer2LC*) chains significantly improved locomotor function. The locomotor effects of the canonical antioxidant genes were in accord with their effects on longevity; CAT (b, *UAS-Arctic Aβ*_*1–42*_*UAS-CAT*) and mitSOD2 (b, *UAS-Arctic Aβ*_*1–42*_*UAS-mitSOD2*) improved climbing, whereas SOD1 (b, *UAS-Arctic Aβ*_*1–42*_*UAS-SOD1*) accelerated the decline. The antioxidant genes had similar effects when co-expressed with wild-type Aβ_1–42_ with co-expression of ferritin heavy chain (c, *UAS-Aβ*_*1–42*_*UAS-Fer1HC*) and CAT (c, *UAS-Aβ*_*1–42*_*UAS-CAT*) improving locomotor function and SOD1 (c, *UAS-Aβ*_*1–42*_*UAS-SOD1*) accelerating the decline in locomotor function. Co-expression of GST (d, *UAS-Arctic Aβ*_*1–42*_*UAS-GST*) gave a weak rescue of the locomotor deficits and carbonyl reductase (d, *UAS-Arctic Aβ*_*1–42*_*UAS-SNI*) did not significantly improve locomotor function. In control experiments the expression of the key modifiers of Aβ toxicity (FerHC, FerLC and CAT) in the absence of Aβ did not have a beneficial effect on locomotor function at any time-point (supporting Fig. S2) (independent Student’s *t*-test, *P < 0.05, **P < 0.01, ***P < 0.001).

The modifiers were then assessed for their ability to protect against the Aβ_1–42_-mediated decline in locomotor function. Flies co-expressing either ferritin subunit with *Arctic* Aβ_1–42_ were significantly more mobile than flies expressing *Arctic* Aβ_1–42_ alone (Fig. 5a, *elav-Gal4 UAS-Arctic Aβ*_*1–42*_) from day 10 onwards, and both the heavy and light chain flies performed like control flies (Fig. 5a, *elav-Gal4*) up to day 25. The effect of the heavy chain was more potent, maintaining wild-type locomotor performance in *Arctic* Aβ_1–42_ flies to day 35. The behavioural effects of over-expressing the canonical antioxidative genes were in accord with their longevity data; CAT (Fig. 5b, *elav-Gal4 UAS-Arctic Aβ*_*1–42*_*UAS-CAT*) and the mitSOD2 (Fig. 5b, *elav-Gal4 UAS-Arctic Aβ*_*1–42*_*UAS-mitSOD2*) were suppressors of the *Arctic* Aβ_1–42_ locomotor phenotype, whereas the cytoplasmic SOD1 (Fig. 5b, *elav-Gal4 UAS-Arctic Aβ*_*1–42*_*UAS-SOD1*) accelerated the decline in locomotor function. Over-expression of the heavy chain of ferritin (Fig. 5c, *elav-Gal4 UAS-Aβ*_*1–42*_*UAS-FerHC*) and CAT (Fig. 5c, *elav-Gal4 UAS-Aβ*_*1–42*_*UAS-CAT*) had similar beneficial effects in flies expressing wild-type Aβ_1–42_ (Fig. 5c, *elav-Gal4 UAS-Aβ*_*1–42*_). As expected, flies co-expressing SOD1 (Fig. 5c, *elav-Gal4 UAS-Aβ*_*1–42*_*UAS-SOD1*) with wild-type Aβ_1–42_ demonstrated an accelerated decline in locomotor function. The co-expression of GST (Fig. 5d, *elav-Gal4 UAS-Arctic Aβ*_*1–42*_*UAS-GST*) gave a weak but significant improvement in locomotor function from day 10 onwards. Carbonyl reductase (Fig. 5d, *elav-Gal4 UAS-Arctic Aβ*_*1–42*_*UAS-SNI*) did not significantly improve locomotor function.

### The metal chelator clioquinol reduces β-amyloid peptide-mediated neuronal toxicity and specifically reduces iron levels in the brain

**Fig. 6:**
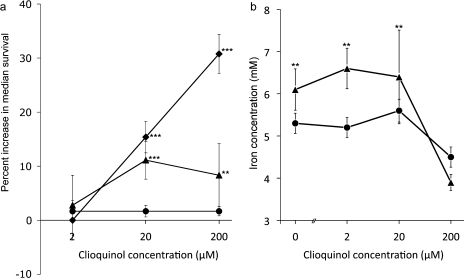
The iron-chelating compound clioquinol was added to the fly food at final concentrations of 2, 20 and 200 μm and its effects on the Aβ-associated longevity phenotype and the accumulation of iron in the fly brain was assessed. The median lifespan of flies expressing Aβ_1–40_ (a, circles) was not significantly increased by clioquinol at any concentration; however, flies expressing *Arctic* Aβ_1–42_ demonstrated a clear concentration-related increase in median survival (a, diamonds). Wild-type Aβ_1–42_ flies exhibited an intermediate, but significant, response (a, triangles). Kaplan–Meier survival statistics with the log rank test were used to analyse the data (significance of difference from no-clioquinol control, **P < 0.01, ***P < 0.001, unlabelled *P* > 0.05). Flies expressing Arctic Aβ_1–42_ (b, triangles) accumulated significantly more iron in their brains than control flies (b, circles) and this was only reversed by treatment with 200 μm clioquinol. Error bars show the SD (*n* = 3). The significance of the difference between clioquinol-treated and non-treated flies was calculated pairwise using the two-tailed Student’s *t*-test (**P < 0.01).

That the protection against Aβ toxicity afforded by ferritin is mediated in part by the chelation of Fe^2+^ and Fe^3+^ ions was investigated by treating flies expressing Aβ_1–40,_ wild-type Aβ_1–42_ and *Arctic* Aβ_1–42_ with the metal chelator clioquinol (Kaur *et al.*, 2003) (Fig. 6). Although clioquinol had no effect on the longevity of flies expressing the non-toxic Aβ_1–40_ (Fig. 6a, circles) there was, in contrast, a clear dose-related increase in longevity for *Arctic* Aβ_1–42_ (Fig. 6a, diamonds) flies. Expression of wild-type Aβ_1–42_ (Fig. 6a, triangles) gave an intermediate response to clioquinol treatment with an optimum dose of approximately of 20 μm. The degree of functional rescue in flies expressing *Arctic* Aβ_1–42_ closely reflected the concentration of iron in the extracts of fly heads (Fig. 6b). We found that the presence of *Arctic* Aβ_1–42_ (Fig. 6b, triangles) increased the uptake of iron as compared with control flies (Fig. 6b, circles), a striking effect that is only completely reversed by treatment with 200 μm clioquinol. The levels of zinc (1.5–1.7 mm) were the same in *Arctic* Aβ_1–42_-expressing and control flies and remained unchanged following clioquinol treatment. Copper could not be detected in the head extracts.

### Oxidative damage in flies expressing β-amyloid peptide is reduced by ferritin and increased by superoxide dismutase 1

**Fig. 7:**
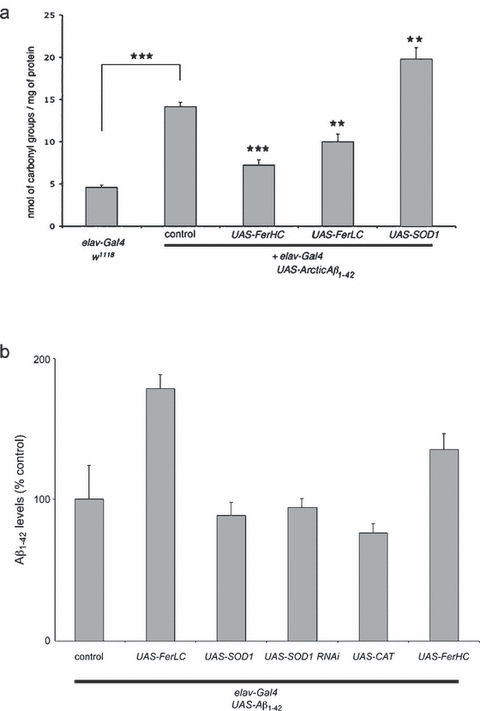
Oxidative stress mediated by the *Arctic* Aβ_1–42_ is reversed by ferritin despite an increase in the levels of Aβ_1–42_. A reduction in carbonyl load was apparent with co-expression of the light chain of ferritin (a, *UAS-FerLC elav-Gal4 UAS-Arctic Aβ*_*1–42*_, *P* < 0.05); however, the effect was more marked following over-expression of the heavy chain of ferritin (a, *UAS-FerHC elav-Gal4 UAS-Arctic Aβ*_*1–42*_, *P* < 0.01). In contrast, co-expression of SOD1 (a, *UAS-SOD1 elav-Gal4 UAS-Arctic Aβ*_*1–42*_, *P* < 0.01) significantly increased the oxidative damage as compared with flies expressing *Arctic* Aβ_1–42_ alone (a, control *elav-Gal4 UAS-Arctic Aβ*_*1–42*_) (**P < 0.01, ***P < 0.001). The concentration of Aβ_1–42_ in the heads of flies was determined for three independent biological replicates for each line of flies. Co-expression of both the heavy and light chains of ferritin results in significantly increased levels of Aβ (b, *UAS-FerLC* and *UAS-FerHC*). In contrast, modification of SOD1 or CAT activity (b, *UAS-SOD1, UAS-SOD1 RNAi* and *UAS-CAT*) has no effect on Aβ levels. Error bars show the SEM.

The quantity of carbonyl groups was assessed in flies expressing *Arctic* Aβ_1–42_ in the presence or absence of the heavy and light chains of ferritin. The expression of *Arctic* Aβ_1–42_ significantly increased the concentration of carbonyl groups (Fig. 7a, *elav-Gal4 w*^*1118*^ vs. *elav-Gal4 UAS-Arctic Aβ*_*1–42*_, *P* < 0.001) but this was reduced by 30% following the co-expression of ferritin light chain (Fig. 7a, *elav-Gal4 UAS-Arctic Aβ*_*1–42*_*UAS-FerLC* and *elav-Gal4 UAS-Arctic Aβ*_*1–42*_, *P* < 0.01). Remarkably, ferritin heavy chain (Fig. 7a, *elav-Gal4 UAS-Arctic Aβ*_*1–42*_*UAS-FerHC*) reduced the carbonyl level (*P* < 0.001) almost to that of control flies (Fig. 7a, control *elav-Gal4 UAS-Arctic Aβ*_*1–42*_). These findings are consistent with ferritin exerting its suppression of the Aβ phenotype by an antioxidant effect. Conversely, the enhancer effect of SOD1 was accompanied by a significant increase in carbonyl levels in *Arctic* Aβ_1–42_ flies (Fig. 7a, *elav-Gal4 UAS-Arctic Aβ*_*1–42*_ vs. *elav-Gal4 UAS-Arctic Aβ*_*1–42*_*UAS-SOD1*, *P* < 0.01). These data suggest that the rapid production of H_2_O_2_ by cytoplasmic SOD1 can overwhelm endogenous CAT resulting in the Fe^2+^-mediated generation of the hydroxyl radical (Fenton reaction) that damages proteins as demonstrated by the increased carbonyl load.

### Ferritin subunits suppress the toxicity of β-amyloid peptide_1–42_ despite increased levels of β-amyloid peptide_1–42_ in the brain

The effect of over-expressing antioxidant transgenes on the level of Aβ_1–42_ in the brains of flies was assessed by an enzyme-linked immunosorbent assay that measured 5 m guanidinium hydrochloride-soluble Aβ_1–42_. Six independent lines of flies expressing *Arctic* Aβ_1–42_ and carrying GS elements that cause over-expression of ferritin subunits were shown to accumulate significantly higher levels of Aβ_1–42_ in their brains (65 pm in control flies vs. 100 pm for GS elements co-expressing the light chain gene, *P* < 0.01, and 141 pm over-expressing the heavy chain gene, *P* < 0.01). This elevation of Aβ_1–42_ when over-expressing ferritin subunits was confirmed in flies co-expressing Aβ_1–42_ with UAS-linked transgenes for either the heavy or light chains (Fig. 7b). In contrast, the levels of Aβ_1–42_ were unaffected by the co-expression of CAT or the over-expression, or the knockdown, of SOD-1. Thus, the rescue mediated by over-expression of oxidative stress genes was not mediated by suppression of the Aβ_1–42_.

## Discussion

Two genome-wide genetic screens were used in our *Drosophila* model of AD to identify genes that play an important role in the toxicity of the Aβ. The first screen, using *Drosophila* cDNA microarrays, quantified the changes in gene transcription that occurred in response to the expression of Aβ_1–42_ as compared with Aβ_1–40_. The second complementary screen, using a library of flies with unique GS element insertions, identified genes, which when transcriptionally up- or downregulated modified the lifespan of flies expressing Aβ_1–42_. Taken as a whole the GS screen was remarkable for the number of genes adjacent to modifying P-elements that had redox or antioxidant activities. Similarly, the cDNA microarray studies showed that redox-associated transcripts were the most robustly represented functional group of genes that were differentially regulated by Aβ_1–42_ expression. This combination of observations is powerful because, whereas the microarray data provide genetic evidence of a response by the brain to oxidative stress, the GS screen, in contrast, points to a direct pathogenic role for oxidative stress in the generation of Aβ-related phenotypes. The role of oxidation in causing disease was further emphasized by the remarkably close correlation between the severity of the phenotypes observed for Aβ_1–40_, Aβ_1–42_ and *Arctic* Aβ_1–42_ and the oxidative modification of brain proteins as determined by carbonyl levels. These findings are concordant with the raised levels of oxidized proteins and lipids in post-mortem AD brains where it is known that oxidative damage is present from the earliest clinical stages of the disease (Nunomura *et al.*, 2001; Markesbery *et al.*, 2005). Our work therefore represents an advance on current clinical work and mouse models that have not clarified whether oxidative stress plays a direct role in the pathogenesis of AD or whether it is a consequence of the disease process. Indeed, the view that oxidative stress is a bystander effect is supported by clinical trial data that show, despite early encouraging data from observational studies (Morris *et al.*, 2002a,b; Zandi *et al.*, 2004) and one prospective trial (Sano *et al.*, 1997), that the antioxidant vitamin E is not able to prevent the onset of dementia or prevent progression of established disease (Petersen *et al.*, 2005).

**Fig. 8:**
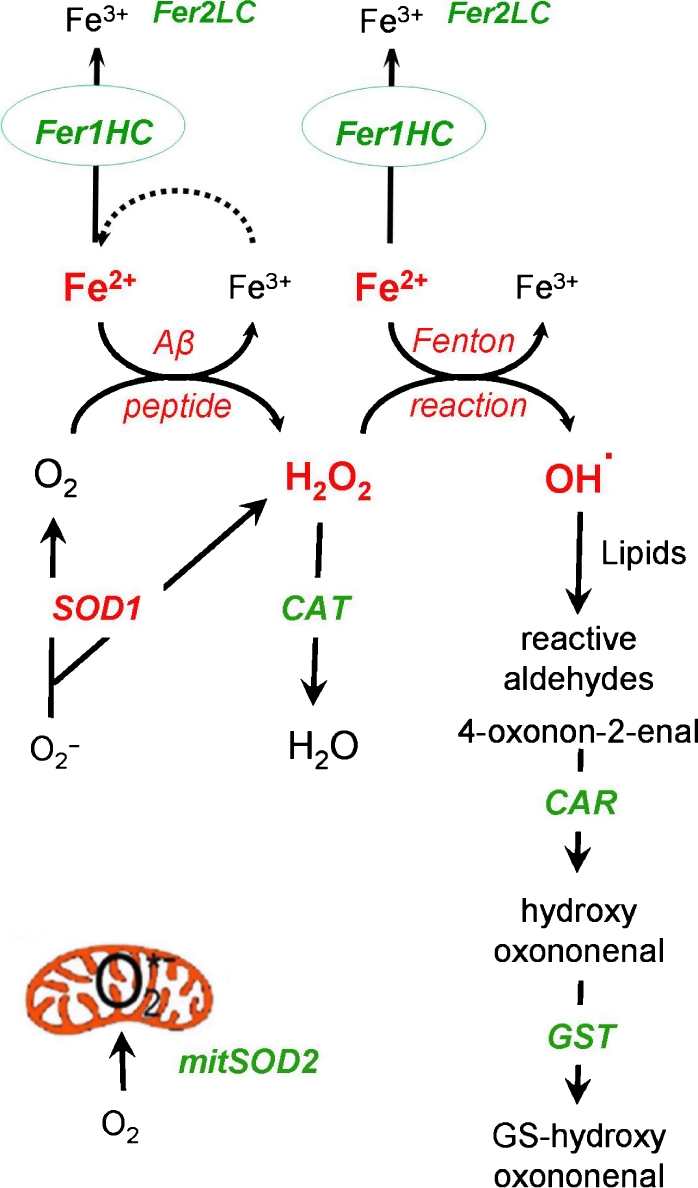
Model of Aβ-mediated oxidative stress. CuZn-SOD1 breaks down superoxide free radicals (O_2_−) in the cytoplasm to produce H_2_O_2_ and molecular oxygen (O_2_). In the presence of oxygen, Fe^2+^ cycles back to Fe^3+^ by the Aβ to produce H_2_O_2_. H_2_O_2_ is neutralized into water by CAT. The heavy chain of ferritin (Fer1HC) has ferroxidase activity, which catalyses the conversion of Fe^2+^ into Fe^3+^ ions. Fe^3+^ is subsequently stored by the light chain of ferritin (Fer2LC). Thus, ferritin has two effects: it prevents Fe^2+^ from interacting with the Aβ and producing H_2_O_2_ and it prevents Fe^2+^ from reacting with H_2_O_2_ and producing the free radical hydroxyl (OH^.^) via the Fenton reaction. Hydroxyl radicals can also oxidize lipids to generate long-lived reactive aldehydes. Carbonyl reductase (CAR) and GST are downstream antioxidant defences that participate in the detoxification of the reactive aldehyde species.

In this work we have undertaken a molecular dissection of the oxidative stress pathway (Fig. 8) in order to gain a more detailed understanding of which oxidative species were of particular importance in Aβ-mediated neurodegeneration. This work has emphasized three main sources of oxidative stress: firstly, the generation of hydroxyl radicals via the Fenton reaction (Smith *et al.*, 1997; Dikalov *et al.*, 2004), secondly, mitochondrial superoxide levels and thirdly, reactive aldehyde species produced by lipid oxidation. Although each of these sources contributes significantly to oxidative stress, the conversion of H_2_O_2_ to the hydroxyl radical is the most important toxic process. The most immediate support for this comes from the profound protection afforded to flies when CAT is co-expressed with Aβ_1–42_ because hydroxyl radical generation will predictably be abolished by converting H_2_O_2_ to water.

Only co-expression of ferritin subunits offered a more effective rescue of the longevity phenotype than CAT. Ferritin proteins are highly conserved in evolution and have been extensively characterized (Harrison & Arosio, 1996). Functional ferritin complexes may be composed of various proportions of heavy and light subunits. For instance, in the human, ferritin from the brain is predominantly composed of heavy chain, whereas in the liver the ferritin has a high proportion of light chain. In our model, the over-expression of Fer1HC or Fer2LC occurred in the context of endogenous Fer1HC and Fer2LC expression. Thus, in our transgenic animals we should produce ferritin complexes composed of both subunits but with one or the other representing the major component. Remarkably, ferritin heavy chain was able to restore the longevity and locomotor phenotype of flies expressing the highly toxic *Arctic* Aβ_1–42_ to that of control flies. That this rescue was mediated by an antioxidant effect is supported by the reductions in carbonyl levels in the brains of flies co-expressing either the light or, again more potently, heavy chain of ferritin. It is likely that the sequestration of iron by both ferritin subunits, and the conversion of Fe^2+^ to Fe^3+^ by the heavy chain, slows hydroxyl radical production. Our data show for the first time that it is likely to be the removal of Fe^2+^, by the ferroxidase activity of the heavy chain, that is specifically beneficial. Previous *in-vitro* data have shown that synthetic Aβ can directly generate H_2_O_2_ in the presence of metal ions (Huang *et al.*, 1999a; Tabner *et al.*, 2005) and in particular iron (Khan *et al.*, 2006). Using molecular oxygen as a substrate, the production of H_2_O_2_ by synthetic Aβ depends on Fe^2+^ ions generated via a redox cycling of iron (Huang *et al.*, 1999a; Khan *et al.*, 2006). Thus, it is possible that ferritin can not only prevent the Fenton reaction but additionally the Fe^2+^-scavenging activity of ferritin heavy chain can also protect neurones against the intrinsic redox properties of Aβ (Fig. 8).

The powerful antioxidant properties of ferritin can also rescue the locomotor deficits associated with Aβ expression despite an accompanying twofold increase in Aβ load. These data make the modulation of brain iron metabolism an attractive therapeutic target not least because the concentration of Fe^2+^ in amyloid plaques is 1 mm, almost three times the normal level (Bush, 2003). Furthermore, clioquinol, a metal chelator, reduces plaque deposition in mouse models of AD (Cherny *et al.*, 2001), is safe in clinical trials (Ritchie *et al.*, 2003) and, as we show here, specifically reduces iron levels in the brain and prolongs the survival of flies expressing Arctic Aβ_1–42_.

Oxidative stress has been linked with mitochondrial dysfunction in several neurodegenerative disorders including AD and also Parkinson’s and Huntington’s diseases (Bowling & Beal, 1995; Maier & Chan, 2002). In AD there is evidence that soluble oligomeric aggregates of Aβ may damage the insulating properties of the neuronal plasma membrane (Kayed *et al.*, 2004) resulting in calcium influx (Demuro *et al.*, 2005). Mitochondria from patients with AD are impaired in their ability to buffer calcium influxes and also show defects in their respiratory complexes, particularly complex IV, resulting in increased generation of reactive oxygen species (Sheehan *et al.*, 1997; Abramov *et al.*, 2004). Our data are consistent with these hypotheses showing that over-expression of the mitochondrial superoxide scavenging enzyme mitSOD2 offered modest but significant protection against Aβ toxicity.

It was surprising that the co-expression of SOD1 enhanced the toxicity of Aβ_1–42_. This result underlines the low toxicity of the superoxide anion and again emphasizes the importance of H_2_O_2_ in the oxidative stress pathway. By showing that dominant negative mutants of SOD1 prolong the life of the AD flies, our findings demonstrate that SOD1 catalytic activity mediates its toxicity. Furthermore, RNAi knockdown of SOD1 protein expression did not provide any additional rescue, indicating that our data are not confounded by toxicity caused by SOD1 misfolding and aggregation as is proposed for SOD1-linked familial amyotrophic lateral sclerosis (Lynch *et al.*, 2004). The finding of elevated carbonyl levels in the brains of flies co-expressing SOD1 and *Arctic* Aβ_1–42_ suggests that rapid dismutation of superoxide can overwhelm the capacity of endogenous CAT to remove the consequent H_2_O_2_. In addition, the dismutation of superoxide radicals releases molecular oxygen that may become a substrate for the Aβ-dependent production of H_2_O_2_ (Fig. 8). Nevertheless, the enhancer activity of SOD1 in *Drosophila* was unexpected because previous studies in a mouse model of AD showed that SOD1 knockdown caused increased activation of apoptotic pathways (Chen *et al.*, 2005). Further work is required to determine whether SOD1 upregulation is toxic in a mammalian model of AD.

Finally, we have evaluated the effect of two enzymes that are involved in the detoxification of reactive aldehydes (GST and carbonyl reductase). Reactive aldehydes, such as 4-oxonon-2-enal, result from hydroxyl radical-mediated lipid oxidation and accumulate in the brains of patients with AD (Lovell *et al.*, 1997; Markesbery & Lovell, 1998). Despite being less reactive than the hydroxyl radical, the greater stability of the aldehydes permits damage to proteins and DNA over longer times and distances within the cell. We have shown that over-expression of GST or carbonyl reductase gave only a modest rescue of Aβ toxicity, suggesting that lipid damage is indeed downstream of the most important toxic events.

In summary, we have used genetic screens and a candidate gene approach to dissect the contribution of reactive oxygen species to the toxicity of Aβ in our *Drosophila* model of AD. We have found that the primary oxidative stressors are likely to be H_2_O_2_ and the consequent hydroxyl radical. Preventing oxidative stress, specifically by manipulating iron metabolism, provides a powerful strategy for reducing Aβ toxicity in AD.

## Abbreviations

Aβ: β-amyloid peptide
AD: Alzheimer’s disease
CAT: catalase
Fer1HC: ferritin 1 heavy chain
Fer2LC: ferritin 2 light chain
GS: Gene Search
GST: glutathione-S-transferase
mitSOD2: mitochondrial Mn-superoxide dismutase 2
SNI: *Sniffer*
SOD: superoxide dismutase
UAS: upstream activating sequence

## Supporting Information

Fig. S1. (a) Experimental design for the Affymetrix chip analysis of differential gene transcription.

Fig. S2. In control experiments, the expression of the key modifiers of A toxicity in the absence of A did not have a beneficial effect on locomotor function at any time point.
