# Supplementary material for: Fenton chemistry and oxidative stress mediate the toxicity of the β-amyloid peptide in a Drosophila model of Alzheimer’s disease
Source: Eur J Neurosci. 2009 Apr;29(7):1335–47. doi: 10.1111/j.1460-9568.2009.06701.x (PMC2777252; doi:10.1111/j.1460-9568.2009.06701.x)
Supplement: Supplementary file 1 [file ejn0029-1335-SD1.doc]

**Figure S1**

a) Experimental design for the Affymetrix chip analysis of differential gene transcription.


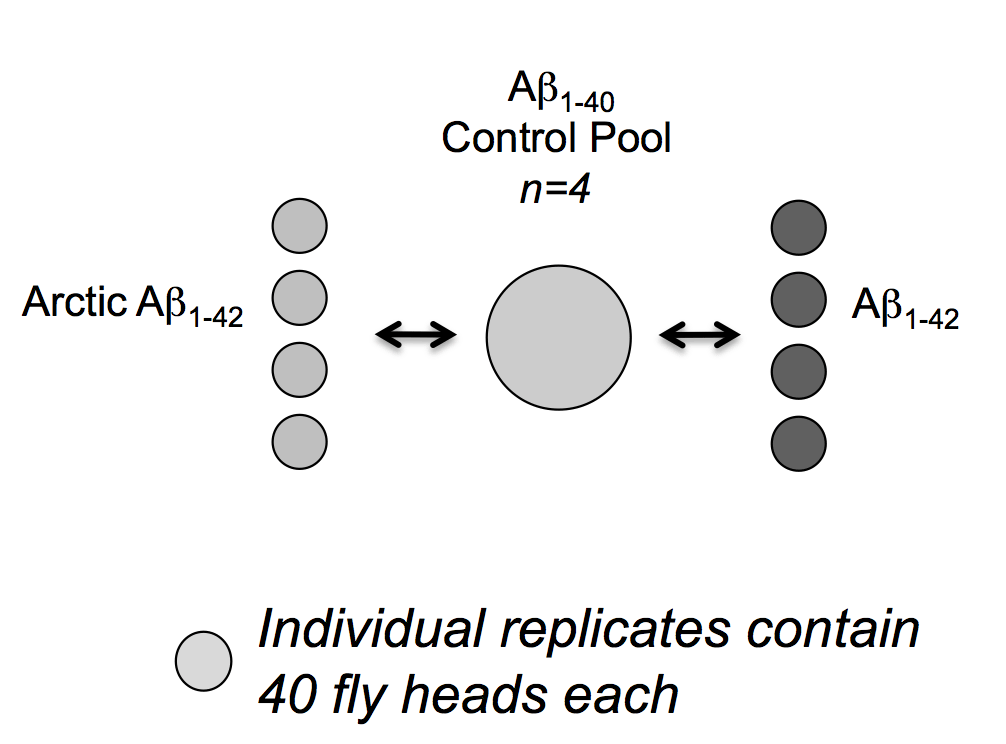


b) Analysis of functional enrichment in genes that are significantly differentially regulated between experimental conditions.

Key:

O = the observed number of genes in the category that are differentially expressed (p<0.01).

E = the expected number of differentially expressed genes in the category assuming that the effects were random.

R = the ratio of enrichment for the category

P = the significance of enrichment in the category

Entries in bold have a role in redox reactions.

**Aβ_1-42_ vs. Abeta40 day 0**

By biologicial process

G1/S transition of mitotic cell cycle: O=2;E=0.15;R=13.33;P=0.0086165734451514

monosaccharide metabolism: O=6;E=1.62;R=3.7;P=0.0053546205907352

cellular lipid metabolism: O=11;E=4.7;R=2.34;P=0.0068495257919063

phospholipid metabolism: O=5;E=1.2;R=4.17;P=0.0066242589917795

di-\, tri-valent inorganic cation transport: O=4;E=0.65;R=6.15;P=0.0037779198728774

calcium ion transport: O=4;E=0.44;R=9.09;P=0.00084466682151395

metal ion transport: O=7;E=2.15;R=3.26;P=0.0053996584457564

immune system process: O=8;E=2.42;R=3.31;P=0.0027250999641981

response to stimulus: O=23;E=13.74;R=1.67;P=0.007858067284412

defense response: O=15;E=6.66;R=2.25;P=0.0023218013349788

response to toxin: O=6;E=1.64;R=3.66;P=0.005703540666339

By molecular function

inositol or phosphatidylinositol phosphatase activity: O=3;E=0.26;R=11.54;P=0.0018287384137115

glucosidase activity: O=3;E=0.45;R=6.67;P=0.0096100793427749

calcium channel activity: O=3;E=0.41;R=7.32;P=0.0072222791668605

voltage-gated calcium channel activity: O=2;E=0.15;R=13.33;P=0.0089053749934181

**Aβ_1-42_ vs. Aβ_1-40_ day 3**

By biologicial process

G1/S transition of mitotic cell cycle: O=2;E=0.14;R=14.29;P=0.008301077535546

alcohol catabolism: O=4;E=0.85;R=4.71;P=0.0096911076320296

monosaccharide catabolism: O=4;E=0.85;R=4.71;P=0.0096911076320296

hexose catabolism: O=4;E=0.85;R=4.71;P=0.0096911076320296

glucose catabolism: O=4;E=0.85;R=4.71;P=0.0096911076320296

hexose metabolism: O=5;E=1.3;R=3.85;P=0.0093066436388087

cellular lipid metabolism: O=11;E=4.61;R=2.39;P=0.0059295974587778

membrane lipid metabolism: O=5;E=1.32;R=3.79;P=0.00993298225165

phospholipid metabolism: O=5;E=1.18;R=4.24;P=0.0061102157235466

di-\, tri-valent inorganic cation transport: O=4;E=0.64;R=6.25;P=0.0035246715568472

calcium ion transport: O=4;E=0.43;R=9.3;P=0.000785543695172

metal ion transport: O=7;E=2.11;R=3.32;P=0.0048591830944439

immune system process: O=7;E=2.38;R=2.94;P=0.0092718817077911

defense response: O=14;E=6.53;R=2.14;P=0.0050938233081434

response to toxin: O=6;E=1.61;R=3.73;P=0.0051938683638217

By molecular function

inositol or phosphatidylinositol phosphatase activity: O=3;E=0.25;R=12;P=0.0017336316144395

glucosidase activity: O=3;E=0.44;R=6.82;P=0.0091341647899928

**oxidoreductase activity: O=18;E=8.75;R=2.06;P=0.0023416079438473**

**dioxygenase activity: O=2;E=0.08;R=25;P=0.0025583837556971**

**oxidoreductase activity\, acting on single donors with incorporation of molecular oxygen\, incorporation of two atoms of oxygen: O=2;E=0.08;R=25;P=0.0025583837556971**

**oxidoreductase activity\, acting on CH-OH group of donors: O=6;E=1.83;R=3.28;P=0.0095764865046404**

**oxidoreductase activity\, acting on the CH-OH group of donors\, NAD or NADP as acceptor: O=5;E=1.24;R=4.03;P=0.0076207142322076**

**oxidoreductase activity\, acting on single donors with incorporation of molecular oxygen: O=2;E=0.08;R=25;P=0.0025583837556971**

calcium channel activity: O=3;E=0.4;R=7.5;P=0.0068606423471316

voltage-gated calcium channel activity: O=2;E=0.15;R=13.33;P=0.0085915622566956

**Aβ_1-42_ vs Aβ_1-40_ day 8**

By biologicial process

Golgi vesicle transport: O=7;E=1.22;R=5.74;P=0.00013561562750265

retrograde vesicle-mediated transport\, Golgi to ER: O=4;E=0.24;R=16.67;P=2.6537693179805E-05

serine family amino acid biosynthesis: O=3;E=0.34;R=8.82;P=0.0034619447123687

serine family amino acid metabolism: O=4;E=0.49;R=8.16;P=0.00091717179328397

L-serine metabolism: O=3;E=0.29;R=10.34;P=0.0020518809590477

protein folding: O=12;E=4.58;R=2.62;P=0.0018571518384245

ATP-dependent proteolysis: O=4;E=0.63;R=6.35;P=0.0027803680826589

protein retention in ER: O=2;E=0.1;R=20;P=0.0024084622421847

carbohydrate metabolism: O=27;E=16.77;R=1.61;P=0.0086296648740162

defense response to fungus: O=4;E=0.63;R=6.35;P=0.0027803680826589

defense response: O=27;E=15.41;R=1.75;P=0.0027264002800371

response to temperature stimulus: O=6;E=1.76;R=3.41;P=0.0071697436221716

response to heat: O=6;E=1.46;R=4.11;P=0.0027908973250811

response to other organism: O=8;E=3.02;R=2.65;P=0.0098099580062282

response to fungus: O=5;E=0.68;R=7.35;P=0.00036836677969902

response to pheromone: O=3;E=0.44;R=6.82;P=0.0077250944453519

By molecular function

signal sequence binding: O=3;E=0.39;R=7.69;P=0.0051601472367105

unfolded protein binding: O=7;E=2.12;R=3.3;P=0.0045822584903502

**oxidoreductase activity: O=31;E=20.04;R=1.55;P=0.0088181598015853 L-iditol 2-dehydrogenase activity: O=2;E=0.14;R=14.29;P=0.0067123140896796**

nutrient reservoir activity: O=3;E=0.24;R=12.5;P=0.0010268104711615

**Arctic Aβ_1-42_ vs Aβ_1-40_ day 0**

By biologicial process

synaptic transmission: O=19;E=10.14;R=1.87;P=0.0055787162382803

regulation of neurotransmitter levels: O=12;E=5.36;R=2.24;P=0.0064902236840678

Wnt receptor signaling pathway: O=7;E=2.33;R=3;P=0.0073567001234983

cell organization and biogenesis: O=82;E=63.76;R=1.29;P=0.0060393964149228

Golgi vesicle transport: O=6;E=1.58;R=3.8;P=0.0037981462905737

retrograde vesicle-mediated transport\, Golgi to ER:

O=3;E=0.32;R=9.38;P=0.0022519825928343

intracellular protein transport: O=36;E=22.93;R=1.57;P=0.0038980900718098

cytoskeleton organization and biogenesis: O=33;E=21.29;R=1.55;P=0.0068944689401961

Golgi organization and biogenesis: O=3;E=0.38;R=7.89;P=0.0042943685313985

acylglycerol biosynthesis: O=2;E=0.13;R=15.38;P=0.0040469422102645

diacylglycerol biosynthesis: O=2;E=0.13;R=15.38;P=0.0040469422102645

acylglycerol metabolism: O=2;E=0.13;R=15.38;P=0.0040469422102645

diacylglycerol metabolism: O=2;E=0.13;R=15.38;P=0.0040469422102645

neutral lipid biosynthesis: O=2;E=0.13;R=15.38;P=0.0040469422102645

neutral lipid metabolism: O=2;E=0.13;R=15.38;P=0.0040469422102645

mRNA processing: O=17;E=9.07;R=1.87;P=0.0085145787231148

nuclear mRNA splicing\, via spliceosome: O=14;E=6.74;R=2.08;P=0.0067772867145944

RNA processing: O=20;E=11.47;R=1.74;P=0.0099021480398258

RNA splicing: O=14;E=6.93;R=2.02;P=0.0086319662814118

RNA splicing\, via transesterification reactions: O=14;E=6.74;R=2.08;P=0.0067772867145944

RNA splicing\, via transesterification reactions with bulged adenosine as nucleophile: O=14;E=6.74;R=2.08;P=0.0067772867145944

transport: O=84;E=64.2;R=1.31;P=0.0032944389447118

protein transport: O=37;E=23.5;R=1.57;P=0.0032670259704047

secretory pathway: O=24;E=9.77;R=2.46;P=2.9774551013123E-05

exocytosis: O=18;E=5.73;R=3.14;P=1.0418288629086E-05

vesicle-mediated transport: O=29;E=13.29;R=2.18;P=4.4794245451953E-05

endocytosis: O=13;E=5.42;R=2.4;P=0.0025507871166356

synaptic vesicle transport: O=9;E=3.59;R=2.51;P=0.0085590973571813

localization: O=101;E=77.37;R=1.31;P=0.001127322349777

establishment of localization: O=97;E=74.34;R=1.3;P=0.001516819672485

establishment of protein localization: O=38;E=23.69;R=1.6;P=0.002061532057175

secretion: O=26;E=10.52;R=2.47;P=1.2425601646817E-05

protein localization: O=40;E=26.21;R=1.53;P=0.0039190042399989

response to stimulus: O=62;E=41.08;R=1.51;P=0.00039427941487608

behavior: O=17;E=9.14;R=1.86;P=0.0091079113280242

defense response: O=33;E=19.91;R=1.66;P=0.0024015124527123

By molecular function

acetylcholine binding: O=4;E=0.55;R=7.27;P=0.0013507074925726

acetylcholine receptor activity: O=4;E=0.55;R=7.27;P=0.0013507074925726

nicotinic acetylcholine-activated cation-selective channel activity:

O=3;E=0.43;R=6.98;P=0.0065858650908785

**Arctic Aβ_1-42_ vs Aβ_1-40_ day 3**

By biologicial process

Golgi vesicle transport: O=6;E=1.5;R=4;P=0.0029712957514045

retrograde vesicle-mediated transport\, Golgi to ER: O=4;E=0.3;R=13.33;P=6.05463082402E-05

Golgi organization and biogenesis: O=3;E=0.36;R=8.33;P=0.003732321584944

alkene catabolism: O=2;E=0.12;R=16.67;P=0.0036647204222982

terpene catabolism: O=2;E=0.12;R=16.67;P=0.0036647204222982

sesquiterpene catabolism: O=2;E=0.12;R=16.67;P=0.0036647204222982

sesquiterpenoid catabolism: O=2;E=0.12;R=16.67;P=0.0036647204222982

juvenile hormone catabolism: O=2;E=0.12;R=16.67;P=0.0036647204222982

terpenoid catabolism: O=2;E=0.12;R=16.67;P=0.0036647204222982

amino acid metabolism: O=21;E=10.86;R=1.93;P=0.0024910610321267

amino acid activation: O=11;E=2.88;R=3.82;P=9.2150683746417E-05

tRNA aminoacylation: O=11;E=2.82;R=3.9;P=7.4973748154208E-05

tRNA aminoacylation for protein translation: O=11;E=2.82;R=3.9;P=7.4973748154208E-05

amino acid and derivative metabolism: O=22;E=12.12;R=1.82;P=0.0043616526498815

aromatic compound metabolism: O=13;E=5.64;R=2.3;P=0.0036989772802903

nucleobase metabolism: O=9;E=3.24;R=2.78;P=0.0043319932922995

purine base metabolism: O=8;E=2.4;R=3.33;P=0.0021729446617295

cellular biosynthesis: O=60;E=34.08;R=1.76;P=4.8979168139797E-06

macromolecule biosynthesis: O=45;E=20.76;R=2.17;P=3.489880209543E-07

protein biosynthesis: O=40;E=19.74;R=2.03;P=9.3501441843773E-06

translation: O=23;E=8.04;R=2.86;P=3.3842399327492E-06

translational initiation: O=9;E=2.64;R=3.41;P=0.00097935192561308

cellular lipid catabolism: O=3;E=0.48;R=6.25;P=0.009548875440164

isoprenoid catabolism: O=2;E=0.12;R=16.67;P=0.0036647204222982

polyisoprenoid catabolism: O=2;E=0.12;R=16.67;P=0.0036647204222982

protein folding: O=14;E=5.64;R=2.48;P=0.0012776612125188

tRNA metabolism: O=11;E=3.9;R=2.82;P=0.0014621848058552

organic acid metabolism: O=27;E=16.32;R=1.65;P=0.0060571881587879

carboxylic acid metabolism: O=27;E=16.32;R=1.65;P=0.0060571881587879

exocytosis: O=13;E=5.46;R=2.38;P=0.0027682592010787

biosynthesis: O=66;E=36.78;R=1.79;P=7.0861792839609E-07

lipid catabolism: O=5;E=0.9;R=5.56;P=0.0013693814092022

nitrogen compound metabolism: O=27;E=16.38;R=1.65;P=0.0063611146739824

response to heat: O=6;E=1.8;R=3.33;P=0.0077357661354268

response to chemical stimulus: O=22;E=11.22;R=1.96;P=0.0016513810242893

response to toxin: O=11;E=4.68;R=2.35;P=0.0063416122777456

response to stress: O=20;E=10.02;R=2;P=0.0021514905255944

By molecular function

macrolide binding: O=2;E=0.17;R=11.76;P=0.0095447211051111

FK506 binding: O=2;E=0.17;R=11.76;P=0.0095447211051111

translation factor activity\, nucleic acid binding: O=11;E=3.4;R=3.24;P=0.00044711618392149

translation initiation factor activity: O=10;E=2.42;R=4.13;P=9.7497722881351E-05

nucleotide binding: O=51;E=36.46;R=1.4;P=0.0068510475273375

ligand-dependent nuclear receptor binding: O=2;E=0.17;R=11.76;P=0.0095447211051111

catalytic activity: O=165;E=144.46;R=1.14;P=0.007877259040664

phospholipase activity: O=5;E=1.21;R=4.13;P=0.0058045685561416

hydrolase activity\, acting on ether bonds: O=3;E=0.29;R=10.34;P=0.0017341324435357

ether hydrolase activity: O=2;E=0.12;R=16.67;P=0.0033747371200717

leukotriene-A4 hydrolase activity: O=2;E=0.12;R=16.67;P=0.0033747371200717

epoxide hydrolase activity: O=2;E=0.12;R=16.67;P=0.0033747371200717

juvenile hormone epoxide hydrolase activity: O=2;E=0.12;R=16.67;P=0.0033747371200717

ligase activity\, forming carbon-oxygen bonds: O=11;E=2.59;R=4.25;P=3.344420017849E-05

ligase activity\, forming aminoacyl-tRNA and related compounds: O=11;E=2.59;R=4.25;P=3.344420017849E-05

aminoacyl-tRNA ligase activity: O=11;E=2.59;R=4.25;P=3.344420017849E-05

aspartate-tRNA ligase activity: O=2;E=0.17;R=11.76;P=0.0095447211051111

glutamate-tRNA ligase activity: O=2;E=0.17;R=11.76;P=0.0095447211051111

**carbonyl reductase (NADPH) activity: O=2;E=0.17;R=11.76;P=0.0095447211051111**

glycine hydroxymethyltransferase activity: O=2;E=0.17;R=11.76;P=0.0095447211051111

structural constituent of ribosome: O=14;E=6.45;R=2.17;P=0.0046555576231444

translation regulator activity: O=11;E=3.46;R=3.18;P=0.00051998129551961
