# Supplementary material for: Fenton chemistry and oxidative stress mediate the toxicity of the β-amyloid peptide in a Drosophila model of Alzheimer’s disease
Source: Eur J Neurosci. 2009 Apr;29(7):1335–47. doi: 10.1111/j.1460-9568.2009.06701.x (PMC2777252; doi:10.1111/j.1460-9568.2009.06701.x)
Supplement: Supplementary file 2 [file ejn0029-1335-SD2.doc]

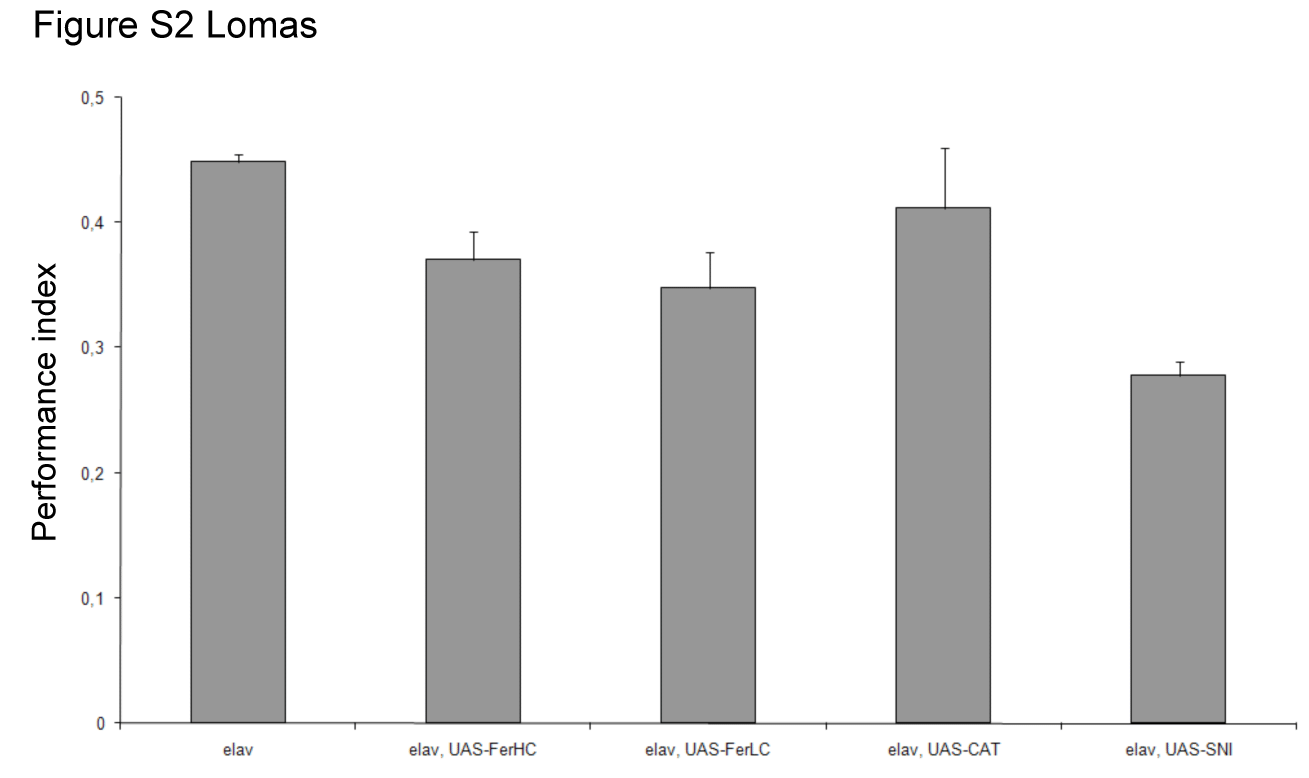


**Figure S2.** In control experiments, the expression of the key modifiers of Aβ toxicity in the absence of Aβ did not have a beneficial effect on locomotor function at any time point. In contrast the locomotor function, as measured by the performance index at day 30, is significantly reduced by expression of ferritin heavy (*UAS-FerHC*) and light chains (*UAS-FerLC*), catalase (*UAS-CAT*) and *Sniffer* (*UAS-SNI*).
